# Long-term safety of once-daily, dual-release hydrocortisone in patients with adrenal insufficiency: a phase 3b, open-label, extension study

**DOI:** 10.1530/EJE-17-0067

**Published:** 2017-03-14

**Authors:** Anna G Nilsson, Ragnhildur Bergthorsdottir, Pia Burman, Per Dahlqvist, Bertil Ekman, Britt Edén Engström, Oskar Ragnarsson, Stanko Skrtic, Jeanette Wahlberg, Heinrich Achenbach, Sharif Uddin, Claudio Marelli, Gudmundur Johannsson

**Affiliations:** 1 Department of EndocrinologySahlgrenska University Hospital and Institute of Medicine, Sahlgrenska Academy, University of Gothenburg, Gothenburg, Sweden; 2 Department of EndocrinologySkåne University Hospital Malmö, University of Lund, Lund, Sweden; 3 Department of Public Health and Clinical MedicineUmeå University, Umeå, Sweden; 4 Departments of Endocrinology and Medical and Health SciencesLinköping University, Linköping, Sweden; 5 Department of Medical SciencesEndocrinology and Metabolism, University Hospital, Uppsala, Sweden; 6 AstraZeneca R&DMölndal, Sweden; 7 Shire International GmbHZug, Switzerland; 8 ShireLexington, Massachusetts, USA

## Abstract

**Objective:**

To investigate the long-term safety and tolerability of a once-daily, dual-release hydrocortisone (DR-HC) tablet as oral glucocorticoid replacement therapy in patients with primary adrenal insufficiency (AI).

**Design:**

Prospective, open-label, multicenter, 5-year extension study of DR-HC conducted at five university clinics in Sweden.

**Methods:**

Seventy-one adult patients diagnosed with primary AI who were receiving stable glucocorticoid replacement therapy were recruited. Safety and tolerability outcomes included adverse events (AEs), intercurrent illness episodes, laboratory parameters and vital signs. Quality of life (QoL) was evaluated using generic questionnaires.

**Results:**

Total DR-HC exposure was 328 patient-treatment years. Seventy patients reported 1060 AEs (323 per 100 patient-years); 85% were considered unrelated to DR-HC by the investigator. The most common AEs were nasopharyngitis (70%), fatigue (52%) and gastroenteritis (48%). Of 65 serious AEs reported by 32 patients (20 per 100 patient-years), four were considered to be possibly related to DR-HC: acute AI (*n* = 2), gastritis (*n* = 1) and syncope (*n* = 1). Two deaths were reported (fall from height and subarachnoid hemorrhage), both considered to be unrelated to DR-HC. From baseline to 5 years, intercurrent illness episodes remained relatively stable (mean 2.6–5.4 episodes per patient per year), fasting plasma glucose (0.7 mmol/L; *P* < 0.0001) and HDL cholesterol (0.2 mmol/L; *P* < 0.0001) increased and patient-/investigator-assessed tolerability improved. QoL total scores were unchanged but worsening physical functioning was recorded (*P* = 0.008).

**Conclusions:**

In the first prospective study evaluating the long-term safety of glucocorticoid replacement therapy in patients with primary AI, DR-HC was well tolerated with no safety concerns observed during 5-year treatment.

## Introduction

Adrenal insufficiency (AI) is a potentially fatal rare disease that requires lifelong glucocorticoid replacement therapy (1), and long-term clinical outcomes with standard treatments remain unsatisfactory (2, 3, 4, 5, 6). Patients with primary AI have a mortality risk of more than double that of the general population, and quality of life (QoL) is impaired compared with that in healthy controls (4, 7, 8). A recent prospective study also suggests that QoL continues to decline over time in patients with AI receiving conventional treatment (9).

Conventional glucocorticoid replacement with hydrocortisone or cortisone acetate needs to be administered multiple times each day to achieve adequate cortisol levels, resulting in plasma cortisol peaks and troughs throughout the day (1). Glucocorticoid overexposure is associated with altered sleep and increased cardiometabolic risk, particularly when cortisol levels are elevated in the late afternoon and evening (1, 10, 11, 12, 13, 14, 15, 16). On the other hand, underexposure is associated with an increased risk of adrenal crisis (AC), and insufficient replacement therapy during intercurrent illness places the patient at risk of a life-threatening AC (13). Current data suggest that the incidence of AC remains unacceptably high despite optimization of conventional, immediate-release hydrocortisone and patient education (17). As long-term side effects and AC are major concerns (18, 19), avoiding glucocorticoid overexposure and underexposure continues to be a major challenge in the management of AI patients (5).

A once-daily, dual-release hydrocortisone (DR-HC) tablet, comprising an immediate-release coating and an extended-release core, has been developed for oral glucocorticoid replacement therapy in AI (20). Clinical trials have demonstrated that DR-HC replacement resembles the daily normal cortisol profile more closely than conventional hydrocortisone replacement (20, 21, 22). In addition, a 3-month randomized, controlled, two-way crossover, clinical trial conducted in patients with primary AI showed that DR-HC treatment was associated with significant improvements in cardiometabolic factors and QoL compared with conventional hydrocortisone tablets administered three times per day (TID) (22). Other studies have shown that glucose homeostasis and serum lipid profiles are improved when switching from conventional replacement to DR-HC treatment and that body mass index (BMI) and waist circumference are reduced (9, 23).

The primary objective of the current study was to investigate the long-term safety and tolerability of DR-HC in patients with primary AI, including assessments of glucose and lipid metabolism, blood pressure, body weight and QoL.

## Subjects and methods

### Study design

This was an open-label, multicenter, phase 3b, long-term extension study conducted at five university clinics in Sweden with a follow-up period of 5 years (EudraCT number: 2008-003990-42). Enrolled patients were either newly recruited or they had completed a randomized, 3-month crossover study of once-daily DR-HC vs hydrocortisone TID plus a 6-month open-label extension period of DR-HC (referred to as the 9-month study hereafter) (22). Details of the 9-month study have been reported previously (22).

After enrollment into the long-term extension study (baseline), patients underwent full clinical examination, which included medical history, physical examination, vital signs and laboratory parameters. All patients returned to the clinic every 6 months. Newly recruited patients also underwent a telephone assessment at 4 weeks and returned to the clinic at 3 and 6 months after enrollment. At clinic visits, patients had a full clinical examination, study drugs were dispensed, and patient questionnaires were collected. Participants were monitored throughout the study for the occurrence of adverse events (AEs).

### Study participants

Male and female patients were eligible for inclusion if they were aged 18 years or more, had been diagnosed with primary AI at least 6 months previously and had been receiving stable doses of glucocorticoid replacement therapy (total dose: 15–40 mg/day) for at least 3 months prior to study entry. Key exclusion criteria have been described previously (24).

All patients provided signed informed consent, and the study protocol was approved by local ethics committees. The study was conducted according to the principles of Good Clinical Practice and the Declaration of Helsinki.

### Intervention

Patients were instructed to take DR-HC orally in the morning, directly after waking and in the fasting state. The initial dose of DR-HC was the same as the total daily dose of hydrocortisone that they were receiving at enrollment. Dose adjustments could be made according to the treating physician’s judgment based on clinical symptoms and signs associated with glucocorticoid excess or deficiency (25). All patients received oral and written instructions to repeat their daily DR-HC dose once or twice at 6- to 8-h intervals during illness.

### Outcomes

Safety and tolerability of DR-HC was assessed by reporting AEs, episodes of intercurrent illness, increased hydrocortisone need, laboratory parameters (e.g. glucose homeostasis and serum lipids; analyzed at local laboratories) and vital signs. Patients used a diary to record when and why extra doses of hydrocortisone were needed. This allowed increased hydrocortisone need to be categorized due to either intercurrent illness or other reasons (e.g. mental or physical stress). Patient- and investigator-assessed treatment tolerability was evaluated using questionnaires. QoL was evaluated using the Fatigue Impact Scale (FIS) and Psychological General Well-Being (PGWB) questionnaire (26, 27).

### Statistical analysis

All statistical analyses were performed on the safety population, i.e. all patients who received at least one dose of DR-HC. Analyses of AEs, laboratory parameters, vital signs and patient-/investigator-assessed tolerability were also performed for the following subgroups: patients recruited from the 9-month study vs newly recruited patients; patients with diabetes mellitus (DM; defined as type 1 or 2 DM during the 5-year extension study) or who were elderly (defined as aged 65 years or more at baseline) vs the overall population.

Exposure and AE data are presented as descriptive statistics. Exploratory analyses evaluated changes over time (from baseline to 12, 36 and 60 months) in laboratory parameters, vital signs, patient-/investigator-assessed tolerability and QoL, using the Wilcoxon signed-rank test for continuous variables and the Sign test for categorical variables.

## Results

### Baseline demographics and disease characteristics

**Figure 1:**
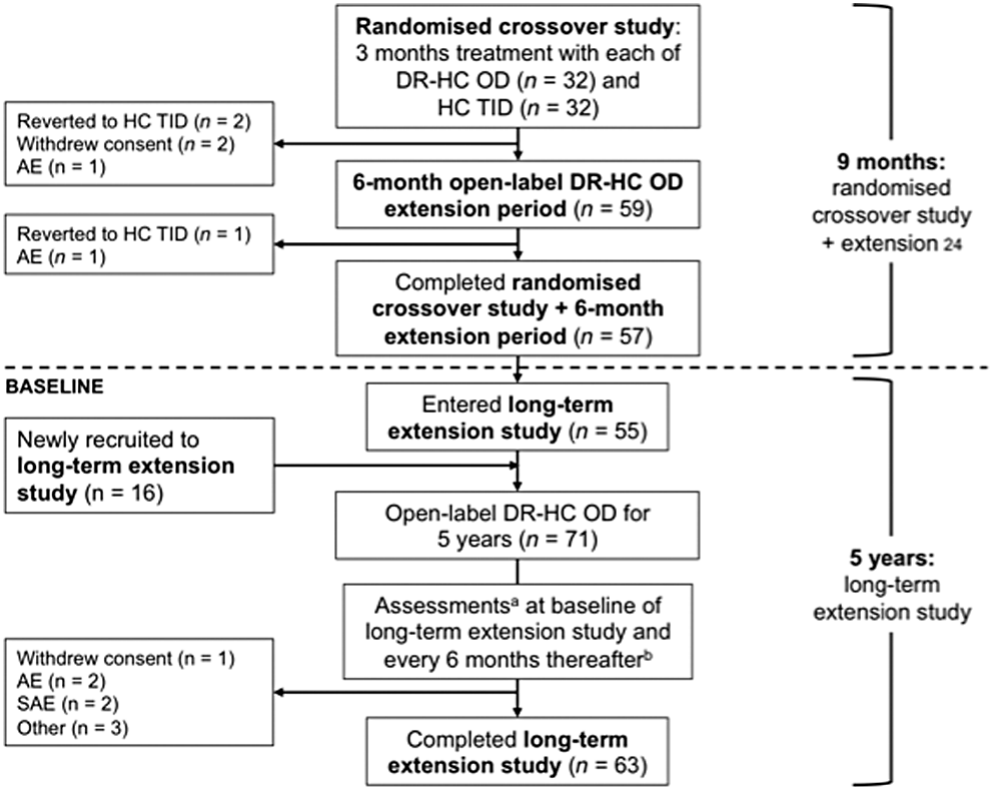
Patient disposition and study flow. AE, adverse event; DR-HC, dual-release hydrocortisone; HC, hydrocortisone; OD, once daily; SAE, serious adverse event; TID, three times daily. ^a^At each assessment, patients had a full clinical examination, study drugs were dispensed and patient questionnaires were collected. ^b^Newly recruited patients had additional assessments at 4 weeks (by telephone) and 3 months (at the clinic).

In total, 71 patients entered the 5-year extension study and were included in the safety population: 55 patients from the 9-month study and 16 newly recruited patients. Sixty-three of 71 patients (89%) completed the 5-year visit. Reasons for study discontinuation were AEs (*n* = 5), serious AEs (*n* = 2) and withdrawal of consent (*n* = 1). Data on the study flow and patient numbers are given in Fig. 1.

Baseline demographics and disease characteristics for the safety population and subgroups are presented in Table 1. Daily hydrocortisone doses at baseline were lower in the newly recruited subgroup than in those recruited from the 9-month study. Hypertension was more common in the DM and elderly subgroups compared with the overall population.

**Table 1:** Demographic and baseline characteristics: safety population. Results are presented as *n* (%) for categorical variables and mean (s.d.) for continuous variables. Percentages are based on the number of patients with non-missing values. For replacement regimen at run-in, the data for once daily include DR-HC use in all patients from the randomized crossover plus 6-month extension study as well as conventional hydrocortisone in newly recruited patients. Diabetes mellitus population is defined as diabetes during the randomized crossover plus 6-month extension study (type 1 diabetes, *n* = 7; type 2 diabetes, *n* = 3; undefined diabetes, *n* = 3; impaired glucose intolerance, *n* = 1). Elderly patients are defined as those who were ≥65 years at inclusion in the randomized crossover plus 6-month extension study.

		Subgroups by recruitment status			
	All patients (n = 71)	From previous study (*n* = 55)	Newly recruited (*n* = 16)	**Patients with DM** (*n* = 14)	**Elderly patients** (≥65 years) (*n* = 11)
Age (year)	48.2 (13.3)	49.0 (13.4)	45.6 (13.1)	56.2 (12.8)	68.3 (2.6)
Sex					
Male	36 (50.7%)	31 (56.4%)	5 (31.3%)	8 (57.1%)	5 (45.5%)
Female	35 (49.3%)	24 (43.6%)	11 (68.8%)	6 (42.9%)	6 (54.5%)
BMI (kg/m^2^)	25.5 (4.1)	25.8 (3.8)	24.5 (4.8)	28.2 (4.7)	26.1 (3.0)
Blood pressure					
Systolic	122.2 (16.5)	122.9 (17.0)	119.5 (14.8)	131.0 (13.0)	139.5 (21.3)
Diastolic	73.9 (8.0)	74.0 (8.3)	73.8 (7.2)	74.3 (7.3)	77.8 (6.8)
Daily dose at run-in (mg)					
20 mg	10 (14.1%)	5 (9.1%)	5 (31.3%)	2 (14.3%)	0 (0)
25 mg	14 (19.7%)	9 (16.4%)	5 (31.3%)	3 (21.4%)	1 (9.1%)
30 mg	33 (46.5%)	30 (54.5%)	3 (18.8%)	6 (42.9%)	9 (81.8%)
35 mg	3 (4.2%)	1 (1.8%)	2 (12.5%)	1 (7.1%)	0 (0)
40 mg	11 (15.5%)	10 (18.2%)	1 (6.3%)	2 (14.3%)	1 (9.1%)
Replacement regimen at run-in					
Once daily	54 (76.1%)	52 (94.5%)	2 (12.5%)	12 (85.7%)	9 (81.8%)
Twice daily	9 (12.7%)	3 (5.5%)	6 (37.5%)	1 (7.1%)	2 (18.2%)
Thrice daily	8 (11.3%)	0 (0)	8 (50.0%)	1 (7.1%)	0 (0)
Duration of AI (years)	16.7 (11.0)	17.5 (11.0)	14.0 (11.0)	20.3 (12.7)	25.5 (11.0)
Diabetes mellitus	14 (19.7%)	11 (20.0%)	3 (18.8%)	14 (100.0%)	4 (36.4%)
Hypertension	16 (22.5%)	14 (25.5%)	2 (12.5%)	7 (50.0%)	5 (45.5%)

AI, adrenal insufficiency; BMI, body mass index; DM, diabetes mellitus.

### DR-HC exposure: maintenance dose and additional use

In the 5-year extension study, total DR-HC exposure was 328 patient-treatment years. Mean total exposure per patient, accumulated over the 5-year study, was 49.7 g of DR-HC, of which 98% was the maintenance dose, 1% was due to intercurrent illness and 1% was increased use not related to intercurrent illness. The total cumulative maintenance dose for each 12-month period of the study remained stable at a mean (s.d.) of 10.2 (2.9) g to 10.6 (2.3) g per patient per year, corresponding to an estimated 28–29 mg/day per patient.

Additional use of hydrocortisone due to intercurrent illness remained stable for the first 4 years (median (range) dose: 95.0 (5.0–850.0)–120.0 (5.0–840.0) mg per patient per year) but was reduced in the fifth year of treatment (60.0 (5.0–815.0) mg). Additional use of hydrocortisone not due to intercurrent illness fluctuated (median (range) dose: 37.5 (5.0–1300.0)–90.0 (0.5–3405.0) mg per patient per year) but there was no trend over time.

Dose adjustments based on the physician’s judgment of individual patient’s needs were made throughout the study (Table 2). Increased daily doses were observed over the 5-year study period in 10 patients receiving 20, 25 or 30 mg at baseline, whereas reductions in daily doses were observed in 13 patients on 25, 30, 35 or 40 mg at baseline. The dose adjustments are presented in Supplementary Figs 1, 2, 3, 4 and 5 (see section on supplementary data given at the end of this article).

**Table 2:** Summary of dose adjustments according to the physician’s judgement of individual patient’s clinical needs.

	**Dose at 5 years** (*n*)					
**Baseline dose**	20 mg/day	25 mg/day	30 mg/day	35 mg/day	40 mg/day	Discontinued
20 mg/day (*n* = 10)	5	3	0	0	0	2
25 mg/day (*n* = 15)	3	7	3	1	0	1
30 mg/day (*n* = 32)	1	3	24	3	0	1
35 mg/day (*n* = 3)	0	1	1	0	0	1
40 mg/day (*n* = 11)	0	1	0	3	4	3

### Long-term safety of DR-HC

Seventy patients (99%) reported 1060 AEs (323 per 100 patient-years). The highest frequency of AEs was recorded in the second year (92.6% of patients), whereas the lowest frequency was recorded in the last year (78.1%). The frequencies in the other years were as follows: year 1, 88.7%; year 3, 83.6% and year 4, 89.1%. Newly recruited patients reported a higher frequency of AEs vs those recruited from the 9-month study (529 vs 273 per 100 patient-years). Compared with the overall population, AE frequency was lower in patients with DM (230 per 100 patient-years) and elderly patients (269 per 100 patient-years).

The most common AEs were nasopharyngitis (70% of patients), fatigue (52%) and gastroenteritis (48%). Most AEs (85%) were considered to be unrelated to DR-HC, whereas 1% and 14% of AEs were assessed by the investigator as probably and possibly related to DR-HC respectively. AEs considered to be probably related to DR-HC were four cases of fatigue and one case each of tachycardia, vertigo, nausea, asthenia, pyrexia, swelling, salt craving and headache. AEs classified as possibly related to cortisol deficiency were reported by 73% of patients over the 5-year period and decreased over time (from 45% in the first year to 23% in the fifth year).

Thirty-two patients (45%) reported 65 serious AEs (20 per 100 patient-years). Of the 65 SAEs, none were considered by the investigator as probably related to DR-HC and four were considered possibly related to DR-HC: acute AI (*n* = 2), gastritis (*n* = 1) and syncope (*n* = 1). The two acute AI SAEs considered to be possibly drug related resulted from infection (sore throat, vomiting and fatigue) and gastroenteritis. Four additional acute AI events (3 SAEs and 1 AE) were reported, all of which were considered by the investigator to be unrelated to DR-HC and were associated with precipitating events (GI illness (*n* = 2), psychological stress (*n* = 1) and dehydration (*n* = 1)). All 6 patients who experienced acute AI recovered. Two SAEs resulted in death (fall from height and subarachnoid hemorrhage), both of which were classified as unrelated to DR-HC.

### Episodes of intercurrent illness and increased hydrocortisone use

There were 709 episodes of intercurrent illness requiring additional hydrocortisone in 64 patients (median 6.0 episodes per patient with any episode) over the 5-year study (Table 3). The median duration per episode was 2.6 days and the median dose of extra hydrocortisone was 19.8 mg per episode. No clear trends over time were observed for number of intercurrent illness episodes, number of days per episode or hydrocortisone dose per episode. However, number of days per episode was the lowest in the fifth year of treatment.

**Table 3:** Episodes of intercurrent illness requiring additional hydrocortisone, over time.

	**0–12 months** (*n* = 71)	**12–24 months** (*n* = 68)	**24–36 months** (*n* = 68)	**36–48 months** (*n* = 67)	**48–60 months** (*n* = 67)	**0–60 months** (*n* = 71)
Intercurrent illness						
Number of episodes	123	132	206	153	95	709
Number of patients with any episode	42	39	38	40	36	64
Number of days with episodes	520	651	473	661	256	2561
Number of episodes per patient						
Mean (s.d.)	2.9 (3.9)	3.4 (5.1)	5.4 (16.7)	3.8 (9.6)	2.6 (3.4)	11.1 (23.8)
Median (range)	2.0 (1−20)	2.0 (1−31)	2.0 (1−104)	1.0 (1−61)	1.5 (1−181)	6.0 (1−169)
Number of days per episode						
Mean (s.d.)	6.5 (19.7)	8.3 (19.1)	3.8 (2.8)	9.1 (28.4)	2.6 (2.6)	4.7 (8.2)
Median (range)	2.8 (1−129)	2.7 (1−113)	3.0 (1−12)	3.0 (1−182)	2.0 (1−16)	2.6 (1−56)
Dose per episode (mg)						
Mean (s.d.)	19.2 (11.4)	18.9 (12.1)	23.5 (13.9)	18.7 (8.9)	19.1 (13.1)	20.5 (11.2)
Median (range)	19.1 (5−60)	18.9 (5−60)	20.0 (5−68)	20.0 (5−40)	20.0 (5−60)	19.8 (5−60)

Mean values do not always consist of 0−60 month values because episodes of extra hydrocortisone use may have overlapped consecutive 12-month periods.

Increased hydrocortisone was used for reasons other than intercurrent illness on 984 occasions in 43 patients (median of 10.0 episodes per patient with any episode) over the 5-year study (Table 4). The median duration was 1.5 days per episode and the median dose of additional hydrocortisone was 13.3 mg per episode. The main reasons for increased hydrocortisone need were physical stress (546 of 984 episodes) and mental stress (339 of 984 episodes). No clear trends over time were observed for number of episodes, number of days per episode or hydrocortisone dose per episode. However, number of days per episode for non-illness-related events was highest in the fifth year of treatment.

**Table 4:** Episodes of increased hydrocortisone use not due to intercurrent illness, over time.

	**0–12 months** (*n* = 71)	**12–24 months** (*n* = 68)	**24–36 months** (*n* = 68)	**36–48 months** (*n* = 67)	**48–60 months** (*n* = 67)	**0–60 months** (*n* = 71)
Increased hydrocortisone use not due to intercurrent illness						
Number of episodes	160	270	212	240	102	984
Number of patients with any episode	26	27	28	23	19	43
Number of days with episodes	302	526	541	444	400	2213
Number of episodes per patient						
Mean (s.d.)	6.2 (8.7)	10.0 (15.0)	7.6 (9.1)	10.4 (16.3)	5.4 (7.7)	22.9 (33.5)
Median (range)	2.5 (1–34)	3.0 (1–62)	2.5 (1–30)	5.0 (1–70)	2.0 (1–34)	10.0 (1–133)
Number of days per episode						
Mean (s.d.)	2.1 (2.1)	1.8 (1.3)	3.4 (6.6)	4.6 (10.9)	5.3 (14.1)	2.2 (1.6)
Median (range)	1.0 (1–9)	1.0 (1–6)	1.2 (1–36)	1.1 (1–51)	1.3 (1–64)	1.5 (1–7)
Dose per episode (mg)						
Mean (s.d.)	15.3 (10.5)	15.5 (15.0)	14.6 (12.3)	13.4 (9.6)	14.5 (10.3)	15.5 (9.6)
Median (range)	10.9 (5–40)	10.0 (1–67)	10.2 (6–60)	10.0 (5–40)	10.0 (5–30)	13.3 (5–40)

Mean values do not always consist of 0- to 60-month values because episodes of extra hydrocortisone use may have overlapped consecutive 12-month periods.

### Laboratory parameters and vital signs

**Figure 2:**
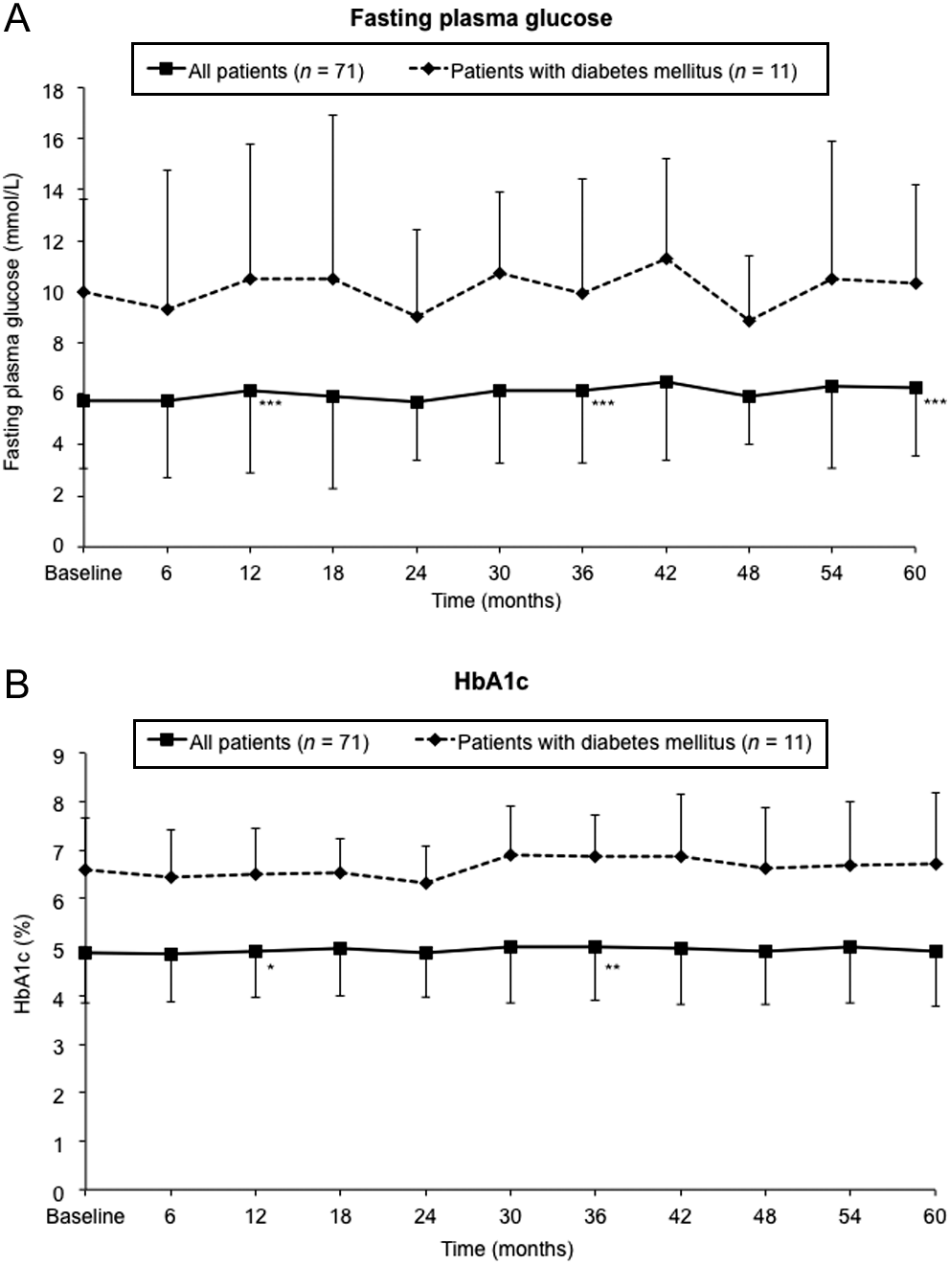
Changes in (A) fasting plasma glucose and (B) HbA1c for the overall safety population and the subgroup of patients with DM, over time. *P < 0.05, **P < 0.01, ***P < 0.0001 vs baseline of the 5-year extension study, analyzed at 12, 36 and 60 months using the Wilcoxon signed-rank test. Data are means (s.d.).

Compared with baseline, small increases in mean fasting plasma glucose (0.4–0.7 mmol/L) were observed at 12, 36 and 60 months (all *P* < 0.001; Fig. 2). There was no significant change in mean HbA1c from baseline to 5 years but small, significant increases were observed at 12 and 36 months (0.6 and 1.1 mmol/mol respectively; *P* < 0.05; Fig. 2). There were no significant changes from baseline to 5 years in mean fasting plasma glucose (1.4 mmol/L; *P* = 0.240) or HbA1c (0.9 mmol/mol; *P* = 0.765) in the subgroup of patients with DM.

**Figure 3:**
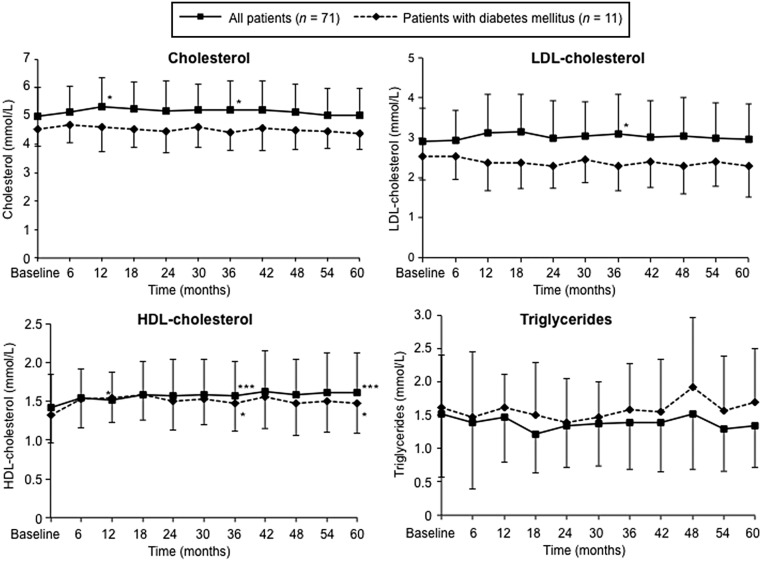
Changes in serum lipids for the overall safety population and the subgroup of patients with DM, over time. DM, diabetes mellitus; HDL, high-density lipoprotein; LDL, low-density lipoprotein. *P < 0.05, **P < 0.01, ***P < 0.0001 vs baseline of the 5-year extension study, analyzed at 12, 36 and 60 months using the Wilcoxon signed-rank test. Data are means (s.d.).

Mean values for total serum cholesterol, LDL cholesterol and triglycerides were similar at baseline and after 5-year treatment with DR-HC in the overall safety population and the DM subgroup (Fig. 3). In the overall safety population, small increases from baseline were observed for total cholesterol at 12 and 36 months (both 0.2 mmol/L; *P* < 0.05), LDL cholesterol at 36 months (0.2 mmol/L; *P* < 0.05) and HDL cholesterol at 12, 36 and 60 months (0.1–0.2 mmol/L; all *P* < 0.05; Fig. 3). Serum lipids generally remained stable throughout the study in patients with DM, although small increases in mean HDL cholesterol were seen at 36 and 60 months (both 0.2 mmol/L; *P* < 0.05).

There were no clinically relevant changes in clinical chemistry variables or hematologic parameters, and there were no statistically significant changes in blood pressure, heart rate, body weight or BMI in the overall safety population or any of the subgroups. Notably, small reductions in systolic blood pressure were seen from baseline to 5 years in all patients (−0.8 mmHg; *P* = 0.974) and patients with DM (−2.9 mmHg; *P* = 0.697); small reductions in diastolic blood pressure were also observed (all patients: −0.8 mmHg; *P* = 0.550; patients with DM: −2.9 mmHg; *P* = 0.209). Body weight remained stable from baseline to 5 years in all patients and the DM subgroup.

### Patient- and investigator-assessed tolerability questionnaires

**Figure 4:**
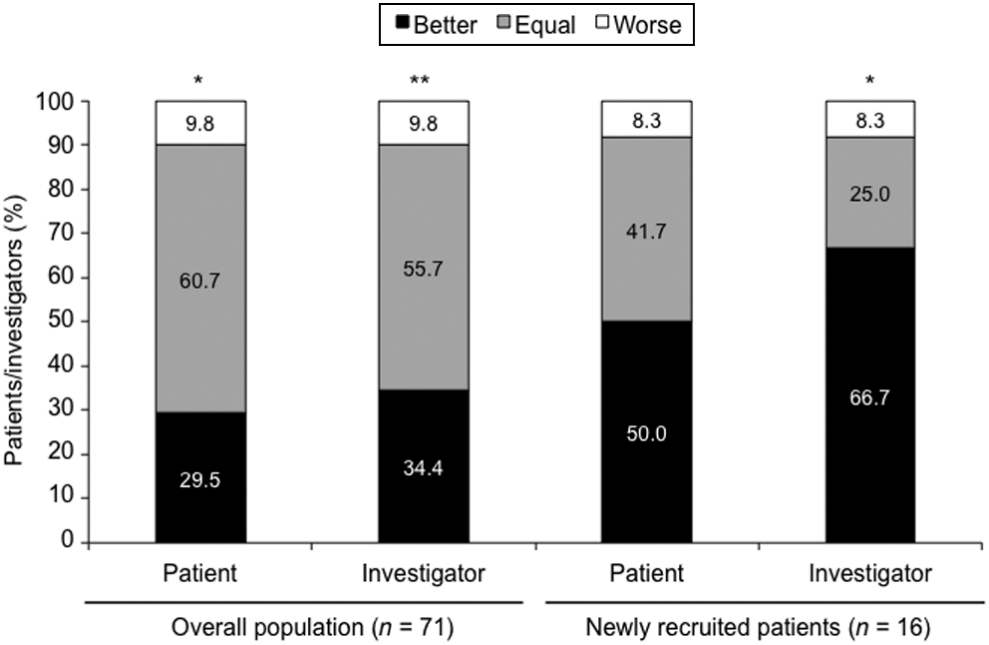
Patient- and investigator-assessed tolerability: changes over time from baseline to 5 years. *P < 0.05, **P < 0.01 vs baseline of the 5-year extension study, analyzed using the Sign test.

Overall, treatment tolerability assessed in patient and investigator questionnaires was significantly improved at 5 years vs baseline (Fig. 4). For newly recruited patients, greater proportions of patients and investigators assessed treatment tolerability to be better at 5 years vs baseline (50% and 67% respectively) compared with the overall population (Fig. 4). There were no significant changes in patient- or investigator-assessed tolerability for the subgroups of DM or elderly patients.

### QoL

**Figure 5:**
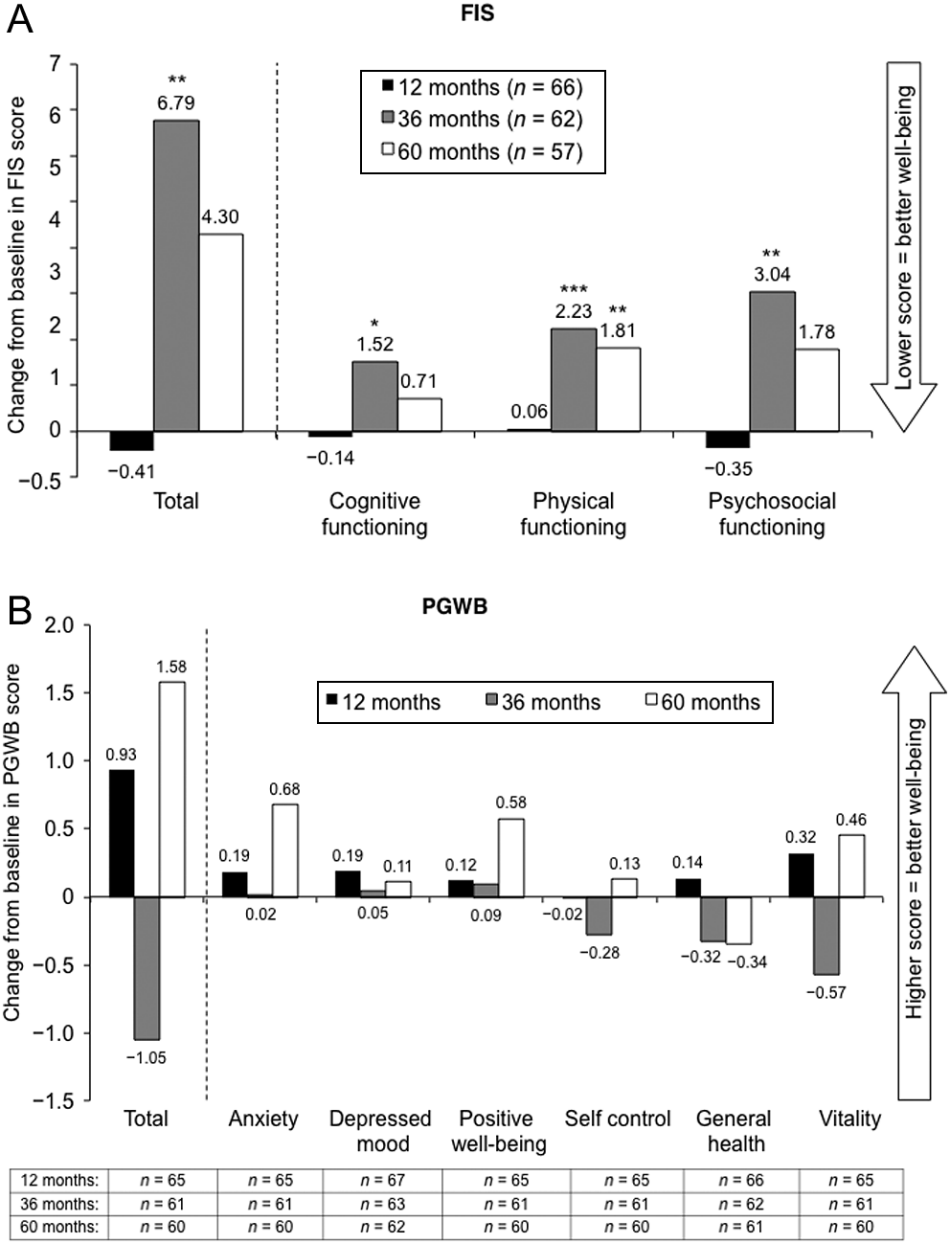
Changes over time from baseline to 12, 36 and 60 months in (A) FIS and (B) PGWB scores. FIS, Fatigue Impact Scale; PGWB, Psychological General Well-Being. *P < 0.05, **P < 0.01, ***P < 0.001 vs baseline of the 5-year extension study, analyzed at 12, 36 and 60 months using the Wilcoxon Signed Rank test.

There were no significant changes in FIS or PGWB total scores from baseline to 5 years (Fig. 5). However, all FIS scores were worse at 3 years and FIS physical functioning score remained worse at 5 years (change from baseline: 1.8; *P* = 0.008). There were no other differences in FIS, and no significant differences in the different domains of PGWB (data not shown).

## Discussion

This is the first prospective, long-term study to investigate the safety and tolerability of glucocorticoid replacement therapy in patients with primary AI. The results demonstrate that the newly developed treatment option, DR-HC, is well tolerated with no safety concerns during at least 5-year treatment in patients with primary AI, including those with DM and elderly patients. The frequency of AEs and SAEs remained stable throughout the 5-year study, no patients died due to AC and the rate of discontinuation was low. Moreover, patient- and investigator-assessed tolerability of DR-HC significantly improved with long-term treatment compared with baseline. These findings expand upon results of an interim analysis of this long-term safety study, which showed that DR-HC was well tolerated for up to 27 months of continuous treatment (24).

The stable safety and tolerability profile seen with DR-HC correlates well with its pharmacokinetic profile showing that within-subject, day-to-day variability in cortisol–time exposure is low with DR-HC treatment (21). The estimated daily maintenance dose of 28–29 mg DR-HC used in this study is somewhat higher than the daily dose of conventional hydrocortisone currently recommended in international guidelines for the management of primary AI (approximately 15–25 mg) (5). However, dosing in the present study is consistent with data from the Swedish Addison Registry of 660 patients, in which the mean hydrocortisone dose was 28.1 mg/day among the 89% of patients who received regular hydrocortisone replacement (28). In addition, there was an overall trend for dose reduction in patients receiving the highest daily doses at baseline (35 and 40 mg) so that dose levels were more in line with clinical practice in the rest of Europe.

To date, this is the only prospective clinical study that has evaluated the management of intercurrent illness episodes over the long term in patients with AI. Results from the preceding 3-month randomized crossover study indicated that DR-HC was at least as effective as hydrocortisone TID in the management of intercurrent illness episodes (22, 24). In the 5-year extension study, the number of intercurrent illness episodes and need for additional hydrocortisone remained stable over 5-year treatment with DR-HC. Importantly, the frequency of AC appears to be lower with long-term DR-HC compared with previous reports in patients with chronic AI treated with conventional glucocorticoids (17, 29, 30), suggesting that intercurrent illnesses were managed effectively. Six acute AI SAEs (ACs), two of which were assessed as possibly drug related, were documented in the present 5-year extension study. This corresponds to 1.8 ACs per 100 patient-years, a markedly lower frequency compared with the 8.3 ACs per 100 patient-years recorded in a large prospective study of patients with AI receiving standard replacement therapy (17). High AC-associated mortality (6%) has also been reported previously (17), but none of the 6 ACs that occurred during DR-HC treatment in the 5-year extension study or the 2 ACs that occurred in the preceding 9-month study (22) resulted in death.

In the preceding 9-month study, DR-HC was associated with favorable effects on cardiovascular risk factors, including blood pressure and glucose metabolism, compared with hydrocortisone TID in the overall population and the subgroup with DM (22). This extension study was not designed to identify small changes in lipids or glucose metabolism, as laboratory analyses were not performed centrally. However, the study does indicate that there is no clear change over time with DR-HC in these parameters, and importantly no deterioration, and no new cases of diabetes. The minimal changes in serum lipids with DR-HC are in contrast to standard glucocorticoid therapy at doses of ≥20 mg/day, which has been shown to be associated with an adverse cardiovascular risk profile (10). The maintenance of stable blood pressure throughout the 5-year study is particularly noteworthy in the elderly and patients with DM who are more likely to have hypertension (31, 32) Indeed, age-dependent changes would likely become evident during this period in DM patients. For example, blood pressure may be expected to increase by approximately 1 mmHg (33, 34) and, in patients with type 2 diabetes, HbA1c has been shown to deteriorate by 0.5% per year (35). However, long-term trends should be interpreted with caution given that there was no control group, the number of subjects was small and changes in antidiabetic, antihypertensive and lipid-lowering medications were not recorded in this study.

The metabolic effects observed in this 5-year study expand upon findings from other clinical studies in patients with AI. Switching from conventional hydrocortisone to DR-HC has been shown to significantly improve glucose and lipid metabolic parameters, central adiposity and QoL vs baseline or continued conventional hydrocortisone (9, 23). The stable metabolic profile observed with DR-HC may be attributed to the particular cortisol release profile of DR-HC that resembles the daily normal cortisol profile more closely than twice- or thrice-daily conventional hydrocortisone (20, 21, 22). Overall, DR-HC appears to provide sustained improvements in cardiovascular risk factors in patients with AI.

Higher doses of conventional hydrocortisone during long-term glucocorticoid replacement have been shown to be associated with worsening QoL (7, 36). By contrast, 10 weeks of treatment with higher doses of hydrocortisone was shown to improve health-related QoL vs lower doses in a recent randomized, double-blind crossover trial in 47 patients with secondary AI (37). In the 3-month comparison with hydrocortisone TID, DR-HC significantly improved total scores and several domain scores on the FIS and the PGWB questionnaire despite an average 20% reduction in cortisol exposure (22). In the 5-year extension study, the physical functioning score on the FIS significantly worsened, but there were trends toward improvement from baseline to 5 years in PGWB total score and several domains. It is possible that the reported worsening of physical functioning over time may reflect the decline in QoL commonly seen with both aging and chronic diseases (38). However, the conflicting results on the two general QoL tools used in this study prevent meaningful conclusions to be drawn about the long-term effects of DR-HC on the QoL of patients with primary AI. When the study was initiated, there were no validated disease-specific QoL instruments for AI but the introduction of the Addison’s Disease-specific QoL questionnaire (AddiQoL) may be useful for future evaluations of QoL in AI (39). Indeed, a recent 12-month study showed that the total score on the 30-item AddiQoL significantly improved after patients with primary AI switched from conventional hydrocortisone to DR-HC (23).

The main limitations of the present study are the uncontrolled, open-label design and the lack of statistical power for comparisons between baseline and subsequent time points. Due to the long-term nature of the study, the 5-year data may not be as robust as those from earlier time points because of the potential for ‘reporting fatigue’. Hence, any statistically significant changes were from exploratory analyses and should be interpreted with caution. Another limitation arises from this being the first prospective study investigating the long-term treatment of primary AI, meaning that there are no other data against which to make valid comparisons. As primary AI is a rare disease, there are few patients available for enrollment in clinical studies, restricting the sample size. Inclusion bias may also affect interpretation, as patients were stable with good QoL at baseline.

In summary, long-term DR-HC therapy for patients with AI was well tolerated with no safety concerns. Intercurrent illness episodes and need for additional hydrocortisone remained relatively stable over at least 5 years of DR-HC treatment and the frequency of AC was lower than that previously reported. The safety surveillance of glucose, lipid and blood pressure did not indicate any cardiometabolic risk with long-term use of DR-HC. This study indicates that DR-HC has the potential to provide effective glucocorticoid replacement therapy for primary AI while avoiding the detrimental cardiovascular and metabolic effects that are common during conventional treatment.

## Supplementary Material

Supporting Figure 1

Supporting Figure 2

Supporting Figure 3

Supporting Figure 4

Supporting Figure 5

## Declaration of interest

A G Nilsson has received speaker support from ViroPharma/Shire. P Dahlqvist has received speaker support from ViroPharma. B Ekman has received consultation fees/honoraria from ViroPharma/Shire. S Skrtic has equity interests in Plenadren and was involved in its development. H Achenbach is an employee of Shire. S Uddin and C Marelli are employees of Shire and hold share/stock options in Shire. G Johannsson has received consultation fees and lecture fees from ViroPharma/Shire. R Bergthorsdottir, P Burman, B E Engström, O Ragnarsson and J Wahlberg have nothing to disclose.

## Funding

This study was funded by ViroPharma (now Shire International GmbH).
