# Supplementary material for: Long-term safety of once-daily, dual-release hydrocortisone in patients with adrenal insufficiency: a phase 3b, open-label, extension study
Source: Eur J Endocrinol. 2017 Mar 14;176(6):715–25. doi: 10.1530/EJE-17-0067 (PMC5425941; doi:10.1530/EJE-17-0067)
Supplement: Supporting Figure 1 [file eje-176-715-s001.pdf]

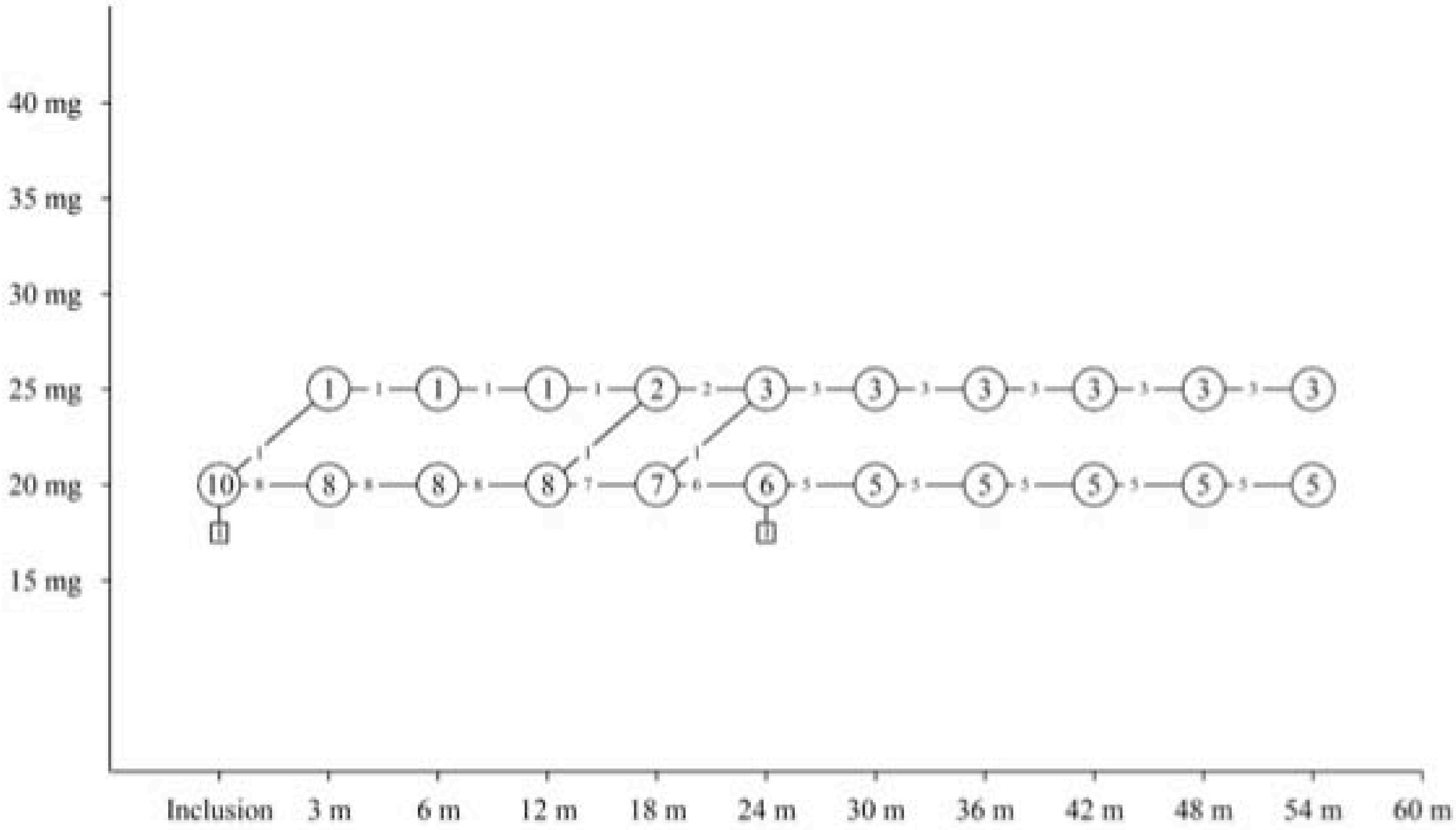

Numbers in circles are number of patients on a certain Plenadren dose (dispension) at the visit
Numbers on lines describes the number of patients that moves from/stays on one dose to another between visits
Numbers in squares are the number of patients that drops out on a certain dose and visit
